# New Glycotoxin Inhibitor from *Sesuvium sesuvioides* Mitigates Symptoms of Insulin Resistance and Diabetes by Suppressing AGE-RAGE Axis in Skeletal Muscle

**DOI:** 10.3390/molecules29153649

**Published:** 2024-08-01

**Authors:** Safina Ghaffar, Rizwana Sanaullah Waraich, Raha Orfali, Areej Al-Taweel, Hanan Y. Aati, Sonia Kamran, Shagufta Perveen

**Affiliations:** 1 Biomedical Research Center, Department of Biomedical & Biological Sciences, Sohail University, Karachi 78400, Pakistanrizwanas.waraich@sohailuniversity.edu.pk (R.S.W.); 2 Department of Pharmacognosy, College of Pharmacy, King Saud University, Riyadh 11495, Saudi Arabia; rorfali@ksu.edu.sa (R.O.); hati@ksu.edu.sa (H.Y.A.); 3 Department of Chemistry, School of Computer, Mathematical and Natural Sciences, Morgan State University, Baltimore, MD 21251, USA; sonia_kamran@yahoo.com; 4 Department of Bacteriology, University of Wisconsin-Madison, Madison, WI 53706, USA

**Keywords:** oxidative stress, inflammation, advanced glycation end products, receptor for advance glycation end products, antidiabetic compound

## Abstract

The current study intended to investigate the role of new natural compounds derived from the *Sesuvium sesuvioides* plant in mitigating symptoms of diabetes and insulin resistance in the diabetic mice model. Anti-advanced glycation activity, insulin, and adiponectin were quantified by enzyme-linked immunosorbent assay (ELISA). Glucose uptake was performed using enzymatic fluorescence assay, and glycogen synthesis was measured using PAS staining. Gene and protein expression was assessed using real time PCR (RT-PCR), and immunoblotting and fluorescent microscopy, respectively. The new flavonoid glycoside eupalitin 3-*O*-α-L-rhamnopyranosyl-(1→2)-β-D-glucopyranoside **1** isolated from *S. sesuvioides* exhibited anti-AGE activity by reducing human glycated albumin in liver cells. In a diabetic mouse model treated with compound **1**, we observed improved glucose tolerance, increased adiponectin levels, and decreased insulin resistance. We also observed alleviated AGEs induced reduction in glucose uptake and restored glycogen synthesis in the compound **1**-treated diabetic mice muscles. Exploring the molecular mechanism of action in skeletal muscle tissue of diabetic mice, we found that **1** reduced AGE-induced reactive oxygen species and the inflammatory gene in the muscle of diabetic mice. Additionally, **1** exhibited these effects by reducing the gene and protein expression of receptor for advanced glycation end products (RAGE) and inhibiting protein kinase C (PKC) delta activation. This further led us to demonstrate that compound **1** reduced serine phosphorylation of IRS-1, thereby restoring insulin sensitivity. We conclude that a new flavonoid glycoside from *S. sesuvioides* could be a therapeutic target for the treatment of symptoms of insulin resistance and diabetes.

## 1. Introduction

It is estimated that almost 537 million adults are living with diabetes. This number is expected to rise to 643 million by 2030 and 783 million by 2045. As per international diabetes federation (IDF) report, Pakistan has the highest comparative prevalence of diabetes (30.8%) in the world. Above 90% of diabetic people have type 2 diabetes, which is caused by demographic, socio-economic, genetic, and environmental factors [1]. A hallmark of modern culture is a preference for processed foods, replete with glycotoxins also known as advanced glycation end products (AGEs) [2,3,4,5].

Glycotoxins are found in various tissues [6,7,8,9] and known to increase oxidative stress and inflammation [10,11], being linked to the recent epidemics of diabetes and various other diseases [12,13,14]. AGEs can cause pancreatic β cell dysfunction, impaired insulin secretion, and insulin resistance, as well as diabetic complications [15,16,17,18]. Consequently, the development of glycotoxin inhibitors is considered to have therapeutic potential in patients with diabetes. Despite extensive research into compounds that could potentially mitigate the effects of glycotoxins, none have yet made it into clinical practice, and therefore there is a need to discover novel anti-glycotoxins.

*Sesuvium* is one of the most pervasive genera of Aizoaceae occurring in many subtropical and tropical regions of the world, including Sudan, Egypt, Africa, Pakistan, and India [19]. Various species belonging to halophytes have been utilized in different conditions: *S. sesuvioides*, Capparis decidua, Achyranthes asper, *Solanum surattense*, and Citrullus colocynthis are used in flu, cold, cough, asthma, and bronchitis [20,21,22]. Previous research has also used *S. sesuvioides* as folk medicine in the local people to treat gout, arthritis, and epistaxis. *S. sesuvioides* was reported for the treatment of endocrine diseases (diabetes and thyroid dysfunction), ulcer, and body pain by local healers (Hakims) or registered traditional practitioners [23,24,25]. *S. sesuvioides* was also used to treat anti-inflammatory disorders like arthritis and gout. *S. sesuvioides* stem and roots, when mixed with water after being stamped, are used for smallpox, chicken pox, measles, hemorrhage, and nose bleeding. A recent in vivo and in vitro study has showed the anti-inflammatory, antipyretic, and analgesic effect of *S. sesuvioides* [23]. The phytochemical constituents of *S. sesuvioides* play a significant role in its diverse biological activities, highlighting its potential for medicinal and pharmacological research. This plant is known to contain several phytochemicals, including gallic acid, benzoic acid, p-coumaric acid, ferulic acid, cinnamic acid, vanillin, umbelliferone, 2-methoxy-4-vinylphenol, and sinapic acid [19]. This study will be the first of its kind to demonstrate the molecular mechanism of novel glycotoxin inhibitor derived from the *S. sesuvioides* plant in improving the pathophysiology of diabetes in diabetic mice. Here, we hypothesized that the compounds isolated from the *S. sesuvioides* plant may reduce the pathophysiology of diabetes by inhibiting glycotoxins. Our study could suggest a potential clinical role for novel glycotoxin inhibitors in the future treatment of diabetes.

## 2. Results

### 2.1. Isolation and Characterization of Compounds from S. sesuvioides

**Figure 1:**
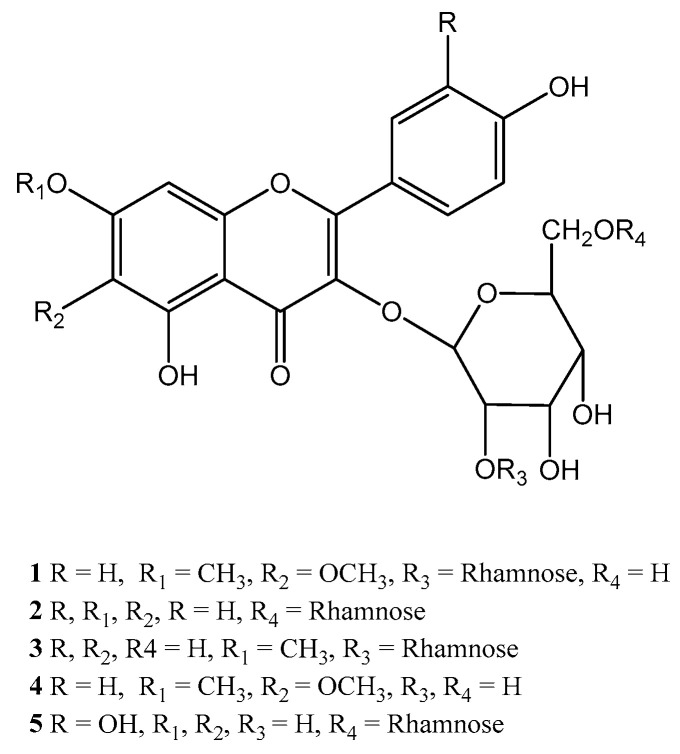
Structures of flavonoid glycosides **1**–**5** isolated from *S. sesuvioides*.

The ethanol extract of the leaves of *S. sesuvioides* was subjected to bioassay-guided fractionation using various chromatographic techniques. It resulted in the isolation of one new (**1**) and four known flavonoid glycosides (**2**–**5**). The structural assessment of these compounds was performed using HRESIMS, ^1^H, and ^13^C NMR data. The assignment of signals was facilitated by COSY, HSQC, and HMBC experiments. The identified compounds were elucidated as kaempferol 3-*O*-α-L-rhamnopyranosyl-(1→6)-β-D-glucopyranoside (**2**), rhamnocitrin 3-*O*-α-L-rhamnopyranosyl-(1→2)-β-D-glucopyranoside (**3**), eupalitin 3-*O*-β-D-glucopyranoside (**4**), and rutin (**5**). These compounds were identified through acid hydrolysis, physical data analysis, and comparison with published literature (Figure 1). Notably, these flavonoid glycosides have not been previously reported in the genus *Sesuvium*. To the best of our knowledge, this is the first documentation of these compounds isolated from this plant.

Compound **1** was isolated as a yellowish gummy solid. Its molecular formula was determined to be C_29_H_35_O_16_, based on the positive mode ESI molecular ion peak [M + H]^+^ at *m*/*z* 639.1948 (calculated C_29_H_35_O_16_: 639.1951) observed in the HRESIMS. Additionally, it exhibited prominent fragments at *m*/*z* 493 and 331, corresponding to the successive losses of rhamnose and glucose sugar units, respectively. The 1H NMR spectrum displayed a signal at δ 12.59 for a chelated hydroxyl group and two singlets at δ 3.89 and 3.71 for the methoxy protons. A C5, C6, and C7 substituted flavonoid skeleton was suggested by the appearance of one-proton singlet at δ 6.85 (s) for the ring A. Two signals of ring B with AA’BB’ pattern at δ 6.90 (d, *J* = 8.5 Hz) and 8.10 (d, *J* = 8.5 Hz) indicated 4’-substituted ring B. Apart from the aglycone protons, two anomeric sugar protons were observed at δ 5.70 (d, *J* = 7.6 Hz) and 5.10 (br s), suggesting the occurrence of two sugar units with β and α configurations, respectively. Further signals of the sugar moieties were observed between δ 4.74 and 3.11. The 1H NMR spectrum further showed a methyl doublet at δ 0.79 (d, *J* = 6.20 Hz), suggesting that one of the sugar residues is rhamnose (Table 1).

The ^13^C NMR spectrum of **1** showed twenty-nine carbon signals comprising three methyl, one methylene, fifteen methine, and ten quaternary carbons. The signals at δ 160.4, 133.1, 178.0, 152.1, and 105.7 were typical of C2, C3, C4, C9, and C10, respectively, of a flavone moiety. Apart from further peaks of the aromatic carbons, the spectrum showed the signals of two anomeric carbons at δ 98.6 and 101.1, oxymethine carbons ranging between δ 68.8 and 78.0 for both sugars, oxymethylene carbons at δ 61.1 for glucoside moiety, and a methyl group of rhamnose sugar at δ 17.7. These data and resistance to the acid hydrolysis suggested that compound **1** is a C3 diglycoside of flavonoid with L-rhamnose at the terminal position while D-glucose is at the internal position. Usually, the oxymethylene carbon of the glucose moiety has the second sugar, but in compound **1**, the up field chemical shift of C6 at δ 61.1 indicated the rhamnose sugar is not present at C6 of the glucose. The point of attachment of the glucose sugar moiety was shown to be at C3 of aglycone through *^3^J* HMBC correlations between the anomeric proton of glucose at δ 5.70 with the C3 at δ 133.1. The point of attachment of rhamnose sugar at the C2 position of glucose moiety was confirmed by *^3^J* HMBC correlations between the anomeric proton of rhamnose at δ 5.10 with the C2 of at δ 78.0. The position of the two methoxy groups on ring A was confirmed by the HMBC experiment in which 6-OCH_3_ proton (δ 3.71) showed a *^3^J* correlation to C6 (δ 132.0) and 7-OCH_3_ proton (δ 3.89) showed a *^3^J* correlation to C7 (δ 159.0). The acidic hydrolysis of 1 yielded an aglycone and rhamnose as the terminal sugar moiety. An MS fragment at *m*/*z* 493.1364 indicated the loss of the rhamnose moiety, and *m*/*z* 331.0823 indicated the eupalitin as the aglycone. The larger coupling constant of anomeric proton of D-glucose sugar (*J* = 7.6 HZ) and singlet of the rhamnose sugar confirmed the β-glucopyranoside and α-L-rhamnopyranosyl sugar configuration. Hence, the structure of compound **1** was defined as the new eupalitin 3-*O*-α-L-rhamnopyranosyl-(1→2)-β-D-glucopyranoside (Figures S1–S6).

### 2.2. Exploring the Effect of Isolated Flavonoid Glycoside on Cell Proliferation

**Figure 2:**
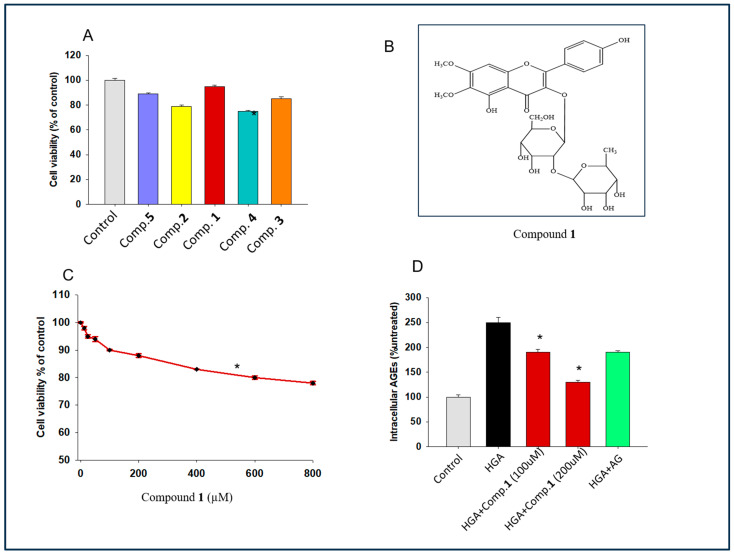
Effect of isolated flavonoid glycoside on cell proliferation. (**A**) Percentage cell viability in human hepatocytes after exposure of cell to isolated flavonoid glycosides (for 24 h) as compared to controls. The data are presented as mean ± SEMs, *n* = 5–8, * *p* < 0.05. Cells stimulated with compounds vs. cells not stimulated. (**B**) The chemical structure of **1**. (**C**) Dose kinetics of **1** in human hepatocytes. (**D**) Intracellular AGE concentration in HepG2 cells as compared to the control. The data are presented as mean ± SEMs, *n =* 5–8, * *p* < 0.05. Cells stimulated with HGA and treated with **1** vs. cells stimulated with HGA alone. Aminoguanidine (AG) was used as a positive control.

Before evaluating the glycotoxin inhibitory effects of the compounds, we determined whether these compounds exhibited any toxicity in vitro. The effect of five isolated compounds from *S. sesuvioides* on cell viability was measured in human hepatocytes (Hep G2 cell line) using the MTT assay. We found that compound **1** exhibited the highest significant cell viability as compared to the control (Figure 2A). The structure of **1** is depicted in Figure 2B. A dose kinetics study was also performed for comp. **1** in hepatocytes. Figure 2C showed that there was no significant impact on cell viability up to 400 µM compared to control cells. For further experiments, we utilized the concentrations of comp.1 that were insufficient to induce cytotoxicity.

### 2.3. Determination of the Effect of Compound ***1*** on Intracellular Glycotoxin Formation

Our subsequent objective was to assess the inhibitory impact of **1** on intracellular advanced glycation end product (AGE) formation. This was accomplished in human hepatocytes by enzymatic fluorescence assay. Our findings revealed a significant decrease in the expression of intracellular AGEs in the cells treated with both compound **1** and human glycated albumen (HGA) as compared to those treated with human glycated albumen alone (Figure 2D).

### 2.4. Determination of the Role of ***1*** against Serum AGEs Levels and HBA1c Levels in Mice

**Figure 3:**
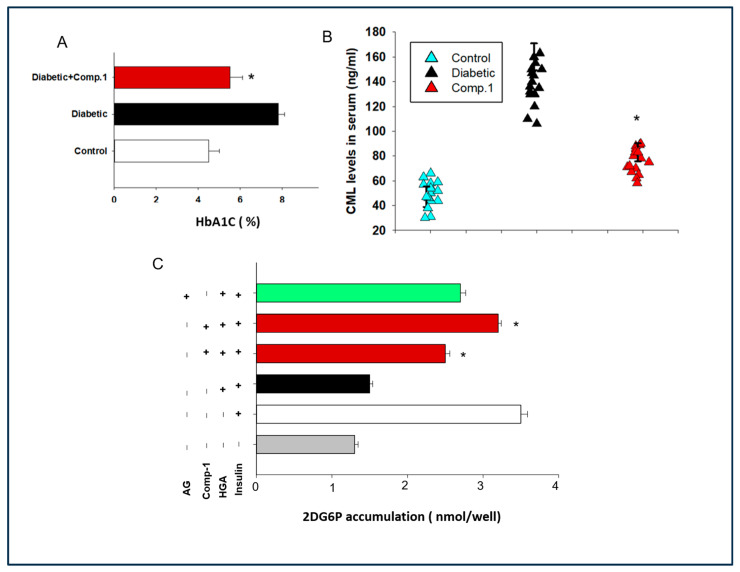
Effect of compound **1** against serum AGE levels, HBA1c levels, and **1** effect on glucose uptake. (**A**) Serum carboxymethyl lysine (CML) levels. (**B**) Serum HBA1c levels. (**C**) In vitro glucose uptake in primary myotubes, treated with either insulin, HGA, **1**, and/or AG. Results represent mean ± SEMs. *n =* 5–10 in each group, * *p* < 0.05. Cell stimulated with insulin, HGA, and compound **1** vs. cells treated with insulin and HGA alone.

The influence of compound **1** in cutting down AGEs concentration in diabetic mice was explored using ELISA. A significant decrease in serum AGEs levels was observed in diabetic mice treated with **1** (Figure 3A). For investigating the relevancy with humans, HBA1c levels were measured in mice blood using an enzymatic assay kit. Diabetic mice treated with 1 exhibited reduced HBA1c levels as compared to untreated diabetic mice (Figure 3B).

To monitor the effect of the anti-AGE compound on glucose uptake in muscle cells of diabetic mice, we measured glucose uptake in primary muscle cells of diabetic mice by enzymatic fluorescence assay [26,27]. By placing 2DG6P standard solutions in wells of the culture plate, a standard curve was generated. We found that treatment with **1** restored AGE-mediated diminished glucose uptake in primary muscle cells of diabetic mice (Figure 3C).

### 2.5. Assessment of Effect of Anti-AGE Compound on Glucose Tolerance and Insulin Secretion in a Mouse Model of Diabetes

**Figure 4:**
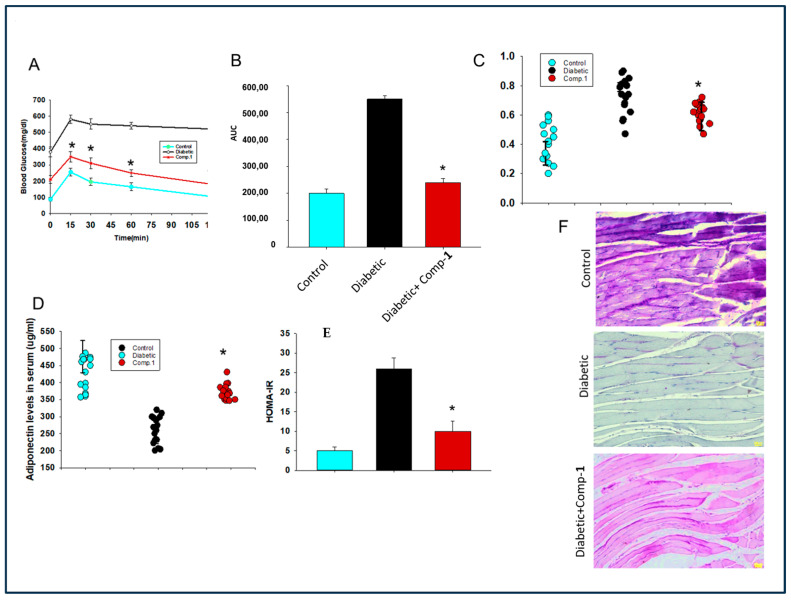
Effect of compound **1** on symptoms of diabetes in a mouse model of diabetes. (**A**) Glucose tolerance. (**B**) Insulin levels. (**C**) Insulin levels. (**D**) Adiponectin levels. (**E**) Insulin resistance. (**F**) Glycogen synthesis in muscles. Periodic acid–Schiff staining of gastrocnemius muscle. Results represent mean ± SEMs. *n =* 10–20 in each group; * *p* < 0.05, diabetic mice vs. treated with **1**.

Next, we addressed the effects of a new anti-glycotoxin compound on hyperglycemia in mice. Mice were tested by the intraperitoneal glucose tolerance test (IPGTT). Substantial improvement in glucose tolerance was observed in diabetic mice treated with compound **1**, demonstrating that **1** addressed hyperglycemic diabetic conditions (Figure 4A,B). Glucose-mediated insulin release from the pancreas was also determined. We observed that there was an increase in insulin secretion in diabetic mice; however, the insulin levels were reduced to normal after treatment with **1** (Figure 4C).

### 2.6. Evaluation of Effect of ***1*** on Insulin Resistance and Hypo-Adiptonectinemia

Further, the role of **1** was verified by measuring insulin resistance in treated diabetic mice by homeostasis model assessment–estimated insulin resistance (HOMA-IR) [28]. Insulin resistance was found to be diminished in the diabetic mice treated with new **1** in comparison to diabetic mice not treated (Figure 4D). As insulin resistance is inversely correlated with adiponectin secretion, we also measured adiponectin levels. We observed that adiponectin hormone levels were significantly enhanced in mice treated with 1, as compared to the diabetic mice (Figure 4E).

### 2.7. Assessment of the Effect of the Anti-AGE Compound on Glycogen Synthesis in Muscle Tissue of Diabetic Mice

The sensitivity of muscle glycogen synthase is also reduced in diabetes and hence its function, causing muscular atrophy. We measured glycogen synthesis using periodic acid–Schiff (PAS) staining in the skeletal muscle of all mice groups. We found that glycogen synthesis was reduced in the muscle of mice with diabetes, while it was restored in mice treated with **1** (Figure 4F).

### 2.8. Deciphering the Molecular Mechanism behind the Role of ***1*** Mediated Improvement in Insulin Resistance

**Figure 5:**
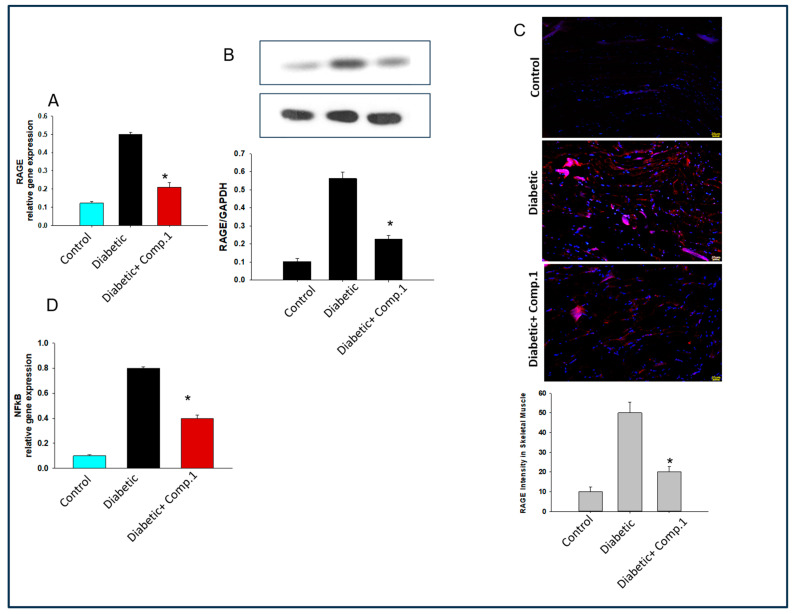

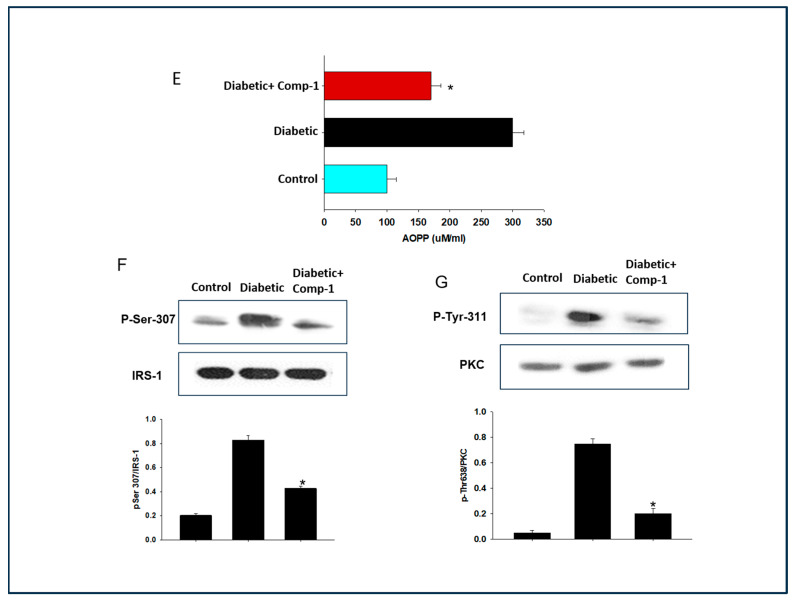
Molecular mechanism behind the role of **1** mediated the improvement in insulin resistance. (**A**) RAGE receptor gene expression. (**B**) RAGE receptor protein expression in muscle. (**C**) RAGE receptor protein expression by immunofluorescence in muscle. (**D**) NF-kappa B gene expression levels in muscle. (**E**) AOPP levels in muscle. (**F**) IRS-1 serine phosphorylation in muscle. (**G**) PKC delta activation in muscle. The data are presented as mean ± SEMs, *n =* 5–10, * *p* < 0.05, diabetic mice vs. diabetic mice treated with **1**.

Various tissues of the body express a receptor for advanced glycation product (RAGE). In light of this, we also determined the expression of the RAGE gene and protein in diabetic mice. A significant fall in RAGE expression was seen in the muscles tissue of **1**-treated diabetic mice (Figure 5A,B). Reduction of RAGE expression by **1** was further verified by immunofluorescence (Figure 5C). AGEs bound to their receptor RAGE also activated oxidative stress and inflammatory cytokines [29]. We found decelerated expression of the NF-kappa B gene in muscle tissues of **1**-treated diabetic mice (Figure 5D). Conversely, oxidative stress was measured as endogenous advanced oxidation protein products (AOPP) production in muscle tissue. AOPP production was significantly downregulated in mice muscle treated with **1** as compared to control mice (Figure 5E).

We further investigated the mechanism of **1** mediating impaired inflammation and oxidative stress, using immunoblotting, since several reports indicate PKC is also involved in enhancing insulin resistance by increasing IRS1 serine phosphorylation. It was observed that serine phosphorylation of IRS-1 was reduced in muscle tissue of diabetic mice treated with **1** as compared to untreated diabetic mice (Figure 5F). We observed activation of PKC delta by Tyr 311 phosphorylation in muscle tissue of diabetic mice while this activation was reduced in mice treated with **1** (Figure 5G).

## 3. Discussion

The aim of this study was to explore the new glycotoxin inhibitors for reducing insulin resistance and symptoms of diabetes. Few studies reported anti-inflammatory effects of *S. sesuvioides* in a number of health conditions [19,24]. However, the present study is the first investigation of the anti-glycotoxin effects of *S. sesuvioides*. We identified a new flavonoid glycoside **1** with potent in vitro and in vivo inhibitory effects against an advanced glycation end product (Figure 2 and Figure 3). Here, we hypothesize that the inhibitor of glycotoxin from *S. sesuvioides* may reduce diabetes symptoms in a mouse model.

In the present report, we observed that **1** administration in a diabetic mouse model could enhance glucose uptake and glucose tolerance (Figure 2 and Figure 3). Such effects could be due to compensated action of hormones from different tissues, including insulin and adiponectin [18,29,30]. Interestingly we observed restoration of levels of insulin and adiponectin levels to that of the control in diabetic mice after treatment with **1** (Figure 4). The most significant result of the study is reduction of insulin resistance in diabetic mice exposed to a new glycotoxin inhibitor from *S. sesuvioides* (Figure 4). Additionally, symptoms of insulin resistance are improved in humans after the restriction of a diet rich in advanced glycation end products [18]. Muscular dystrophy in diabetes could be due to reduced action of insulin action [31,32]. Compound **1** showed restoration of glycogen synthesis in the muscle of diabetic mice (Figure 4). Glycotoxins exert their biological effects in the diabetic muscle using their receptors RAGE [33]. We observed **1** treatment reduced expression of RAGE in the muscle tissue of diabetic mice (Figure 5). Several studies have indicated a RAGE-mediated contribution in inflammation and oxidative stress in diabetes [33]. We have highlighted lower expression of the inflammatory gene and ROS production in the muscle of diabetic mice treated with **1** (Figure 5). Deciphering the molecular mechanism of action, we found RAGE could demonstrate these effects by enhancing activation of PKC. Several other studies also reported the function of PKC in exhibiting RAGE actions [34]. Curran et al. also demonstrated similar results. Evidence indicates that PKC plays a pivotal role in enhancing insulin resistance by increasing serine phosphorylation of IRS-1 [35]. We observed reduced serine phosphorylation in mice treated with a new glycotoxin inhibitor as compared to untreated diabetic mice (Figure 5). Other AGE inhibitors such as aminoguanidine, Alt-486, 2,3-diaminophenazine, and OPB 9195 use various pathways including inhibition of AGE formation and disruption of AGE crosslinking [4]. Our data indicate that compound **1** is an AGE inhibitor, and it uses disruption of glycotoxins with RAGE receptors as a mechanism to inhibit advanced glycation end products.

Limitations of the study include lack of in vivo dose kinetics studies of compound **1** and its comparison with existing advanced glycation end products inhibitors.

The isolation and identification of these compounds from *S. sesuvioides* not only adds to the phytochemical knowledge of this species but also provides a basis for further research into its medicinal potential. Future studies should aim to explore the bioactivities of these compounds in more detail, including their mechanisms of action and potential therapeutic applications. Our research serves as a foundation for correlating the bioactivities of *S. sesuvioides* with its phytochemical constituents, supporting the relevance and importance of this plant in pharmacological studies and natural product research.

## 4. Materials and Methods

### 4.1. General

A MX-500 Bruker spectrometer was used to measure one-dimensional (1D) and two-dimensional (2D) nuclear magnetic resonance (NMR) spectra. The chemical shifts (δ) were calculated (ppm) relative to TMS and J scalar coupling constants reported in Hz. Separations and purifications of secondary metabolites were carried out by using column chromatography either on silica gel 70–230 mesh or RP-18 (E. Merck, Darmstadt, Germany). RP-18 and pre-coated silica gel 60 F_254_ TLC plates were used to check the fractions, and the spots were detected by UV light and by spraying with ceric sulphate and sulfuric acid reagent followed by heating on a hot plate (TLC plate heater III CAMAG, Muttenz, Switzerland). Analytical and reagent-grade solvents were obtained from Sigma-Aldrich (St. Louis, MO, USA). NMR deuterated dimethyl sulfoxide (DMSO-*d*_6_) solvent was purchased from Cambridge Isotope Laboratories (Tewksbury, MA, USA).

### 4.2. Plant Material

The aerial parts of *S. sesuvioides* were collected in the Wadi Laban, Riyadh, Saudi Arabia, in March 2019, and they were identified by the plant taxonomist of the Science College, King Saud University. A voucher specimen (No. 24538) was deposited at the Herbarium of Science College, King Saud University, KSA.

### 4.3. Extraction and Isolation of Compounds from S. sesuvioides

The aerial parts of *S. sesuvioides* (1.2 kg) were extracted by infusing them with 95% ethanol (3 × 4.0 L) at room temperature. The resulting alcoholic extract was dried using a rotary evaporator, yielding 102.5 g of crude gummy extract. This extract was partitioned between water and chloroform (30.0 g), followed by partitioning the aqueous residue with n-butanol (40.5 g). The butanolic fraction was separated by silica (Si) gel column chromatography (CC) eluted with a gradient mixture of CHCl_3_:MeOH (90:10→10:90, *v*/*v*), yielding six major sub-fractions (Fr1-Fr6). Fr1 was further subjected to Si gel CC (590 g, 60 cm × 35 cm) with a gradient elution of CHCl_3_:MeOH (9.5:5→2.0:8.0, *v*/*v*) to afford a fraction that showed one major spot on TLC. This fraction was further recrystallized using MeOH to give compound **1** (11.35 mg). Fr2 was purified by using open glass CC (CHCl_3_:MeOH, 8.5:1.5→2.0:8.0, *v*/*v*) to provide two major subfractions, which were then purified via HPLC by using reverse phase column eluting with H_2_O:MeOH (7.0:3.0) to afford compounds **2** (10.7 mg, Rt 11.3 min) and **3** (15.3 mg, Rt 12.5 min). Fr4 was chromatographed on open glass reversed-phase CC by using Sephadex LH20 with a gradient solvent H_2_O:MeOH (from 9:1 to 2:8) to afford two subfractions, being subjected to further purification via HPLC by using reverse phase column eluting with gradient H_2_O:MeOH (8.0:2.0 to 3.0:7.0) to afford compound **4** (10.7 mg, Rt 11.3 min). Fr5 was chromatographed on Si gel CC with a gradient elution of DCM:MeOH (80:20→30:70, *v*/*v*) to produce white crystalline compound **5** (13.3 mg).

### 4.4. Cell Culture

Cell lines HepG-2 (HB-8065, ATCC, Gaithersburg, MD, USA) were grown in Dulbecco’s modified Eagle’s medium (L0102, Biowest, Mannheim, Germany). Cells were stimulated either with aminogunaidine (AG) (396494, Sigma Aldrich, Gmbh, Wien, Austria) as a positive control and/or human glycated albumen (HGA) (A8301 Sigma Aldrich Wien Austria) and/or new eupalitin 3-*O*-α-L-rhamnopyranosyl-(1→2)-β-D-glucopyranoside (**1**) and insulin (I6634, Merck, St. Louis, MO, USA).

#### 4.4.1. Cell Viability Assay

Human hepatocytes were tested for viability against isolated compounds from *S. sesuvioides* by MTT assay [36]. Cells were treated with five different isolated compounds followed by exposure to tetrazolium dye in a concentration of 0.25 mg/mL. Formazan formed during incubation was dissolved in dimethyl sulfoxide and quantified using a spectrophotometer.

#### 4.4.2. Anti-AGEs Assay

The ability of **1** to reduce intracellular glycation stress was measured by anti-glycation assay, as described [27,29]. The cells were stimulated with Human Glycated Albumin (HGA) (0.1mg/mL) and or **1** for 24 h. Medium was collected and precipitated with absolute trichloroacetic acid (TCA) (87892, Roth, Mannheim, Germany), and after centrifugation, the pellet was washed with 5% TCA and was dissolved in PBS. The suspension was transferred to a 96-well plate, and absorbance was measured at 370nm of excitation and 440 nm of emission using a spectrofluorimeter (Varioskan™ LUX multimode microplate reader, Waltham, MA, USA).

### 4.5. In Vivo Study

Male C57BL/6 mice (Jackson Laboratory, Bar Harbor, ME, USA) were housed in a 12 h light/dark cycle. All experimental procedures were approved by the Sohail university Ethical Review Board (approval#: 2022-000216), in accordance with guide for the care and use of laboratory animals by the National Research Council. The mouse model of type 2 diabetes was developed as described by Gilbert et al. [4]. Mice were assembled into three groups, the control group (mice on normal chow), diabetic group (mice on HFD and STZ-induced diabetes), and the diabetic+ comp. **1** group of diabetic mice treated with compound **1**. Briefly, control mice were fed ad libitum on a standard diet of 10% fat, 63% carbohydrate, 20% protein, 3% fiber, and 1% vitamins and minerals, and the diabetic group and diabetic + comp. **1** group were fed a HFD comprising 60% saturated animal fat, 20% carbohydrate, 20% protein, 3% fiber, and 1% vitamins and minerals for 12 weeks. Mice with HFD were then injected with a low dose of 40 mg/kg STZ intraperitonially for three days. Seven days after STZ injection, mice with fasting blood glucose levels greater than 200 mg/dL were considered diabetic. Diabetic mice were either treated with compound **1** at a dose of 200 µg/kg/day or vehicle for six weeks, intraperitoneally.

#### 4.5.1. Sample Collection

Blood was collected from mice for laboratory experiments. Sera were isolated from the blood accordingly and stored at −80 °C until further assay. Muscle tissue samples for immunofluorescence microscopy were collected in 10% neutral buffered formalin. Muscle tissue was also stored at −80 degrees until use.

#### 4.5.2. Immunological Assays

Cellular and serum AGEs, insulin, and adiponectin levels were measured by the quantitative sandwich enzyme immunoassay technique by using an enzyme-linked immunosorbent assay (ELISA) kit according to the manufacturer’s instructions (STA-817, CML kit, San Diego, CA, USA), Mice insulin ELISA kit, and Mice adiponectin ELISA kit (DRP-300, R&D System, Minneapolis, MN, USA). HBA1c levels were measured using the Mouse hemoglobin A1c assay kit (Crystal Chem, Grove Village, IL, USA). The change of insulin resistance in diabetic mice treated with compound **1** was estimated by applying the homeostatic model assessment of insulin resistance (HOMA-IR) index [29]. The HOMA-IR calculation was performed using the following formula: HOMA-IR = fasting blood glucose (mmol/L) × insulin (μU/mL)/22.5 [29].

#### 4.5.3. Glucose Uptake Assay

The enzymatic fluorescence assay was performed as described using the Glucose Uptake Fluorometric Assay Kit (NA.84, Sigma Aldrich, St. Louis, MO, USA) [28]. Briefly, primary myocytes were isolated from mice quadricep muscle tissue and differentiated into myotubes. Following differentiation, cells were grown in the presence of HGA (0.1 mg/mL) and/or compound **1**. After incubation, the cells were stimulated with insulin (100 nM). Cells were incubated with KRPH buffer containing 1mM 2DG and 0.1% BSA for 20 min at 37 °C in 5% CO_2_. After washing, 25 mL of 0.1 N NaOH was added into the cells. Neutralization was then performed by HCl. The solution was mixed with assay solution (2 to 10 μM resazurin sodium salt, 0.2 units/mL diaphorase, 50 mM KCl, glucose-6-phosphate dehydrogenase, 50 mM TEA, 0.2 units/mL diaphorase, 50 mM KCl, and 0.02% BSA). 2DG6P uptake into the cells was measured by the enzymatic fluorescence assay using a microplate reader (Varioskan™ LUX multimode microplate readerm VL0000D0 + N12391, Thermoscientefic, Waltham, MA, USA).

#### 4.5.4. Periodic Acid–Schiff (PAS) Staining for Glycogen Synthesis

Glycogen synthesis was measured by PAS staining as described by Sultan K. R. et al. [37]. Tissue sections were processed under paraffin and rehydrated. Sections were immersed in periodic acid solution for a minute and washed, followed by 15 min immersion under Schiff’s solution (109033, Merck, Frankfurt, Germany).

#### 4.5.5. Gene Expression by Real-Time PCR

The mRNA expression levels of RAGE were quantified using quantitative PCR (Startagene MX 3000P, Agilent Technologies, Mannheim, Germany), as described previously [30]. Using Trizol reagent (Invitrogen, Foster City, CA, USA), RNA was extracted from the blood. cDNA synthesis was carried out using **1** microgram RNA by the SuperScript III First-Strand Synthesis System (Thermoscientific, Karlsruhe, Germany). Quantitative PCR was performed by SYBR Green Realtime PCR Master Mix (Thermoscientific, Mannheim, Germany) in accordance with the manufacturer’s instructions. Each measurement was performed in triplicate. The mRNA expression levels were normalized to GAPDH (Table S1).

#### 4.5.6. Western Blot

Muscle tissue of all groups of mice was homogenized at 4 °C (Stuart Homogenizer, Leicestershire, UK) in lysis buffer. The tissues were then processed as described previously [38]. Briefly, tissues were homogenized in lysis buffer containing 2.52 mg of protease inhibitor, followed by centrifugation at 16,000 × *g* for 20 min at 4 °C. The supernatant was separated, and protein content was determined by the Bradford method (Figure S7). A total of 100 µg of proteins were separated by SDS-PAGE, and Western blot analysis was performed as described [39] using anti-RAGE, RAGE, ant phosphor serine 307-IRS-1, and anti PKC antibody (Abcam, Cambridge, UK).

#### 4.5.7. Immunohistochemistry

For immunohistochemical staining, sections in different planes from each tissue were double stained for RAGE and nuclei. Sections were deparaffinized, rehydrated, washed in water, and subjected to antigen retrieval in 0.1 m citrate buffer (pH 6.0). They were permeabilized in 0.1% Triton-X-100 in 1x PBS (pH7.2) for 5 min at room temperature and subsequently incubated with blocking solution (2% normal serum in PBS) for 10 min at room temperature. Then, each section was incubated with primary antibody for 1 h. Sections were washed with PBS and stained with secondary antibody for 1 h at room temperature. Counter staining was performed with DAPI. The 3-D Image software (Olympus Stream 2.4) was used to measure fluorescence intensity.

### 4.6. Measurement of Parameters of Oxidative Stress

For measurement of advanced oxidation protein products (AOPP), colorimetry was performed using an OxiSelect™ AOPP Assay Kit (Cell Biolabs Inc., San Diego, CA, USA). Chloramine standard curve was used as a reference. The optical density was read at 340 nm using a multimode microplate reader (Varioskan™ LUX multimode microplate readerm VL0000D0 + N12391, Thermoscientefic, USA).

### 4.7. Statistical Analysis

Statistical analysis was performed using SPSS version 26 software (SPSS Inc., Chicago, IL, USA). Groups of data were compared, after calculating mean +/− SEMs, two-way ANOVA, followed by post hoc analysis (using Dunnett’s multiple comparison tests). Student’s *t*-tests and the chi-squared test were used for additional analysis of variables, when appropriate. A *p*-value ≤ 0.05 was taken to indicate statistical significance

## 5. Conclusions

This is the first report indicating mitigation of diabetes symptoms and its molecular mechanism by a new glycotoxin inhibitor from *S. sesuvioides*. We conclude that compound **1** diminished symptoms of diabetes such as glucose tolerance, glucose uptake, glycogen synthesis, insulin resistance, and hypoadiponectinemia. Another significant finding of the study is reduction in oxidative stress and inflammation in diabetic mice after treatment with compound **1**. The study provided the molecular mode of action of compound **1** by demonstrating reduced serine phosphorylation of IRS-1 through reduced RAGE expression and PKC deactivation. Further studies could be conducted in vivo with compound **1** to decipher its effects in other metabolically active tissues such as the pancreas, liver, fat, kidney, and brain as well. Compound **1** could be a potential glycotoxin inhibitor for clinical use. Additional animal and human studies on compound **1** may reveal its potential as a pharmacological target for the treatment of insulin resistance and diabetes.

## Figures and Tables

**Table 1:** ^1^H- and ^13^C NMR data for compound **1** in DMSO-*d*_6_.

Position	δ_H_ (mult., *J* in Hz)	δ_c_
2	-	160.4
3	-	133.1
4	-	178.0
5	-	152.0
6	-	132.0
7	-	159.0
8	6.85 (s)	91.7
9	-	152.1
10	-	105.7
1′	-	121.2
2′ & 6′	8.10 (d, 8.5)	131.3
3′ & 5′	6.89 (d, 8.5)	115.5
4′	-	156.9
1′	5.70 (d, 7.6)	98.6
2′	3.45 (dd, 8.0, 7.8)	78.0
3′	3.42 (t, 7.8)	77.6
4′	3.76, overlapped	71.0
5′	3.10, overlapped	77.8
6′a	3.56, m	61.1
6′b	3.28, (d, 11.4)	
1′	5.10 (s)	101.1
2′	3.09 (dd, 8.0, 7.8)	70.6
3′	3.47 (t, 9.0)	70.9
4′	3.15, overlapped	72.2
5′	3.75, overlapped	68.8
6′	0.79 (s)	17.7
6-OCH_3_	3.71 (s)	60.5
7-OCH_3_	3.89 (s)	56.9
5-OH	12.60 (s)	-

## Data Availability

Data are contained within the article and Supplementary Materials.

## Supplementary Materials

The following supporting information can be downloaded at https://www.mdpi.com/article/10.3390/molecules29153649/s1. Figure S1. 1H NMR spectrum of compound **1**. Figure S2. 13C NMR spectrum of compound **1**. Figure S3. DEPT 135 NMR spectrum of compound **1**. Figure S4. HSQC NMR spectrum compound **1**. Figure S5. HMBC NMR spectrum compound **1**. Figure S6. Positive HRESIMS of compound **1**. Figure S7. Western Blots images. Table S1: Primers used for real time qPCR.
