# Supplementary material for: New Glycotoxin Inhibitor from Sesuvium sesuvioides Mitigates Symptoms of Insulin Resistance and Diabetes by Suppressing AGE-RAGE Axis in Skeletal Muscle
Source: Molecules. 2024 Aug 1;29(15):3649. doi: 10.3390/molecules29153649 (PMC11314016; doi:10.3390/molecules29153649)
Supplement: Supplementary file 1 [file molecules-29-03649-s001.zip › molecules-3103477-supplementary.pdf]

Supplementary Materials

Figure S1. ^{1}H NMR spectrum of compound **1**.

Figure S2. ^{13}C NMR spectrum of compound **1**.

Figure S3. DEPT 135 NMR spectrum of compound **1**.

Figure S4. HSQC NMR spectrum compound **1**.

Figure S5. HMBC NMR spectrum compound **1**.

Figure S6. Positive HRESIMS of compound **1**.

Figure S7. Western Blots images.

Table S1: Primers used for real time qPCR.

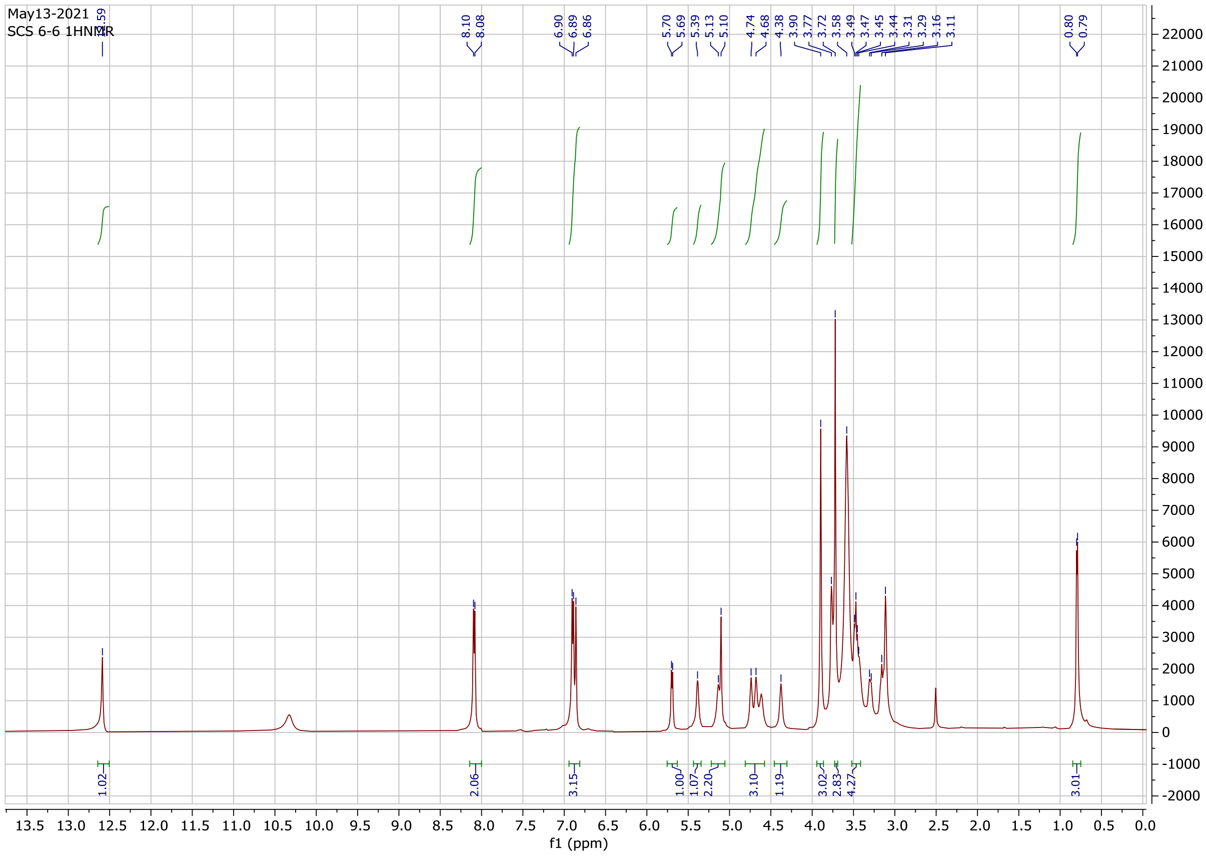

Figure S1. ^{1}H NMR spectrum of compound **1.**

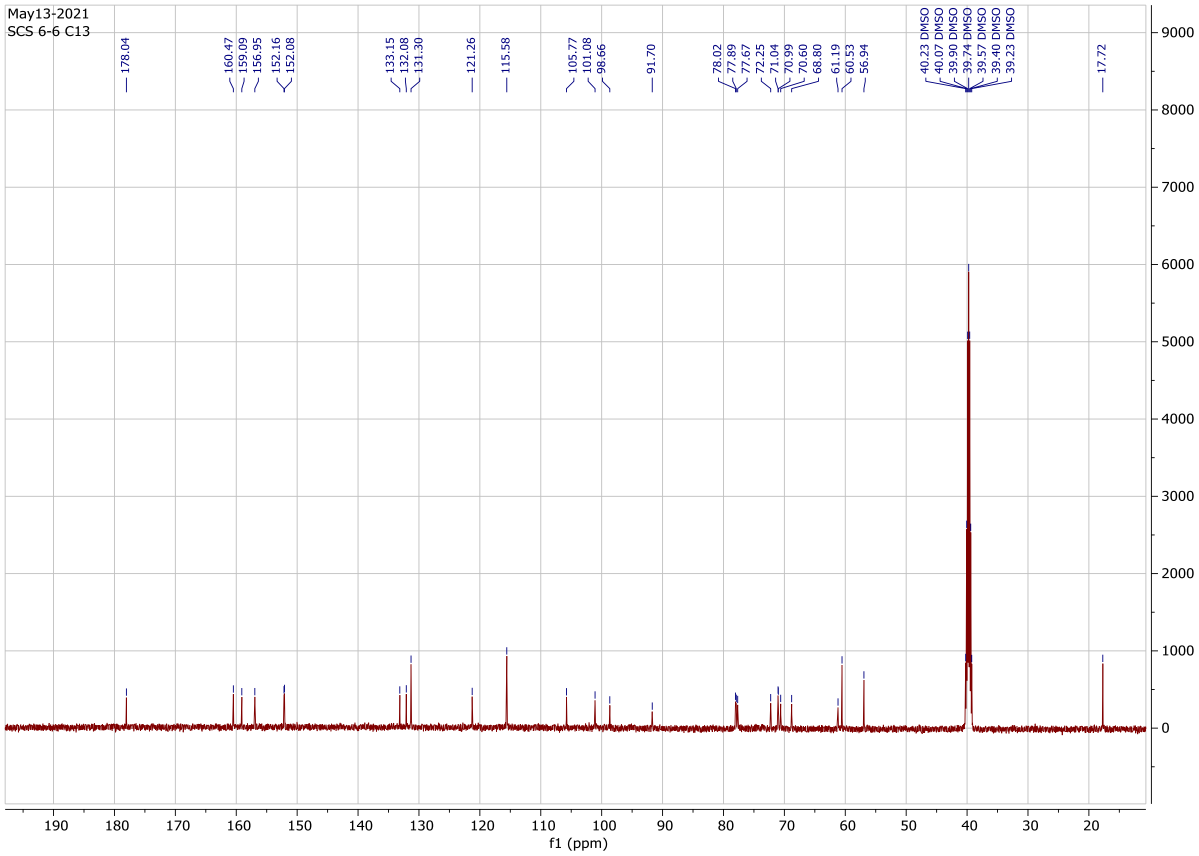

Figure S2. ^{13}C NMR spectrum of compound **1.**

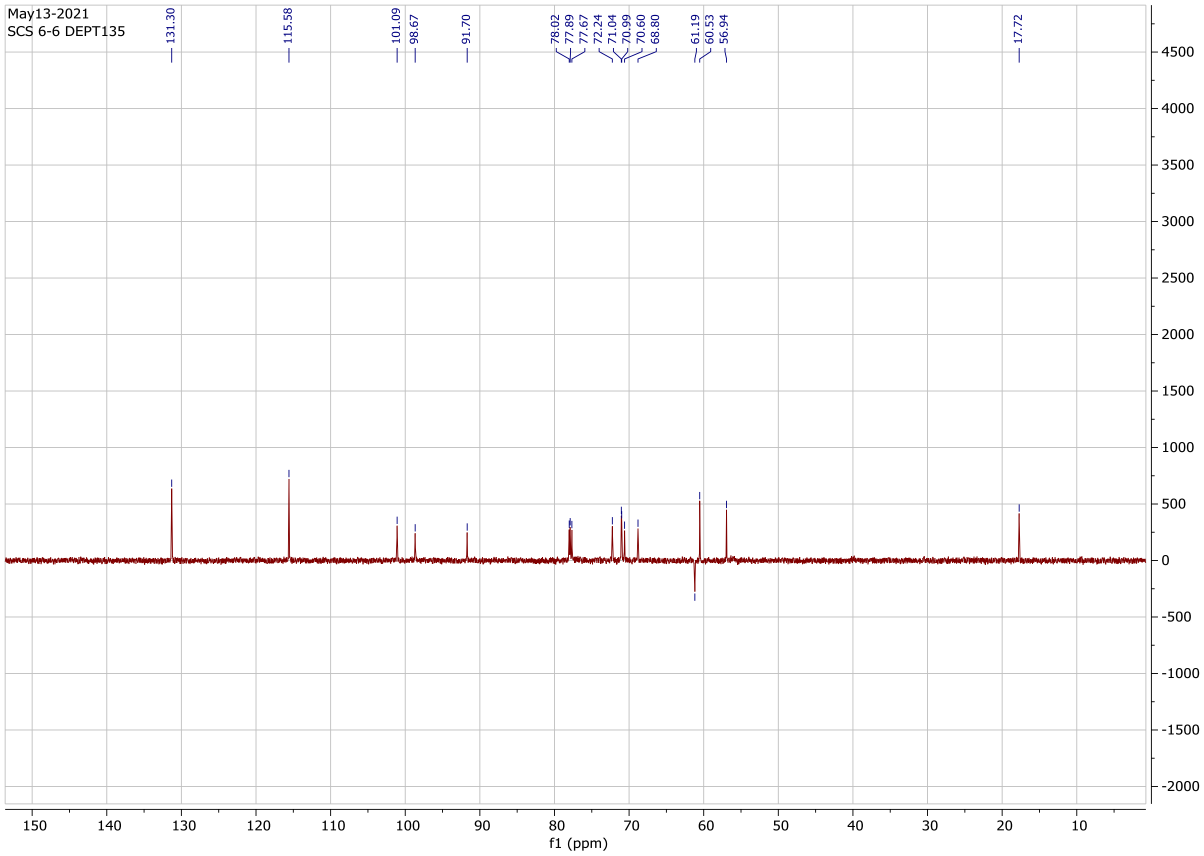

Figure S3. DEPT 135 NMR spectrum of compound **1.**

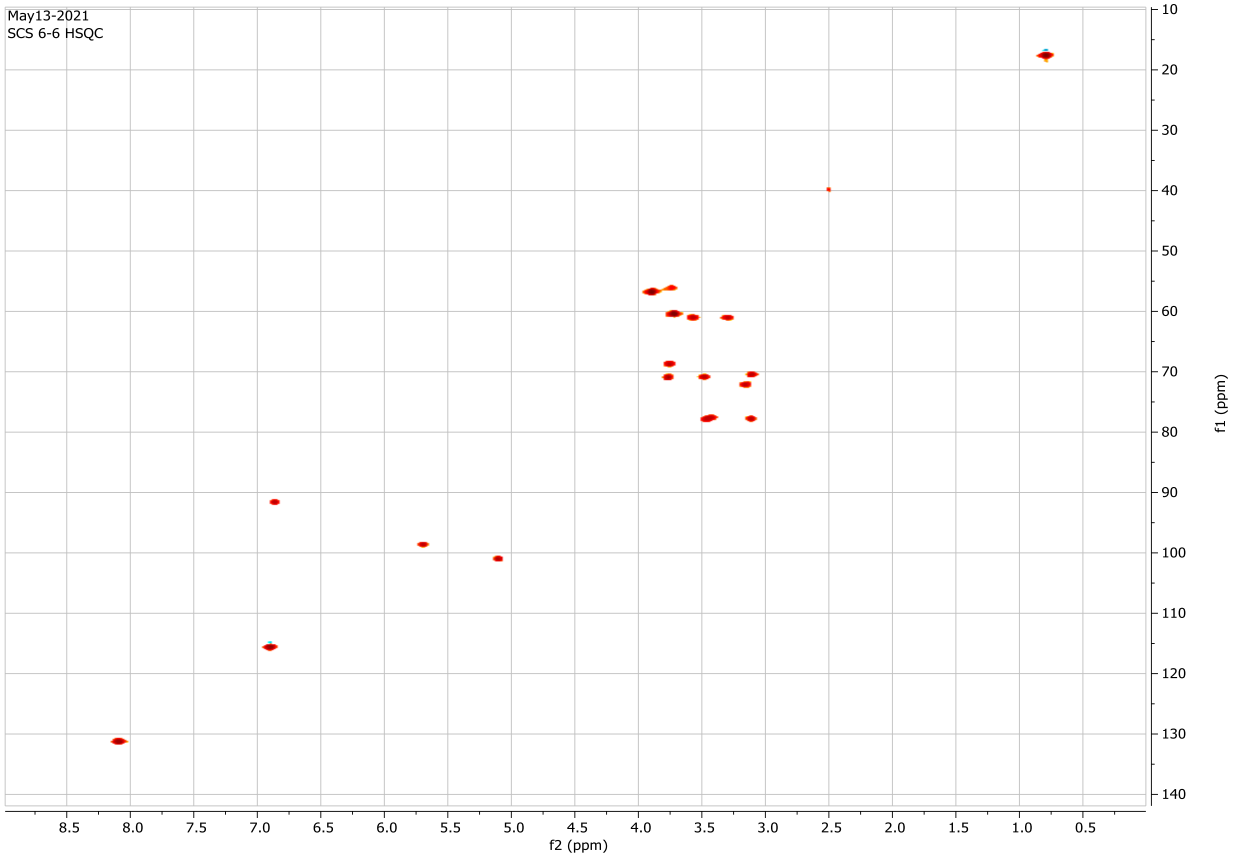

Figure S4. HSQC NMR spectrum compound **1.**

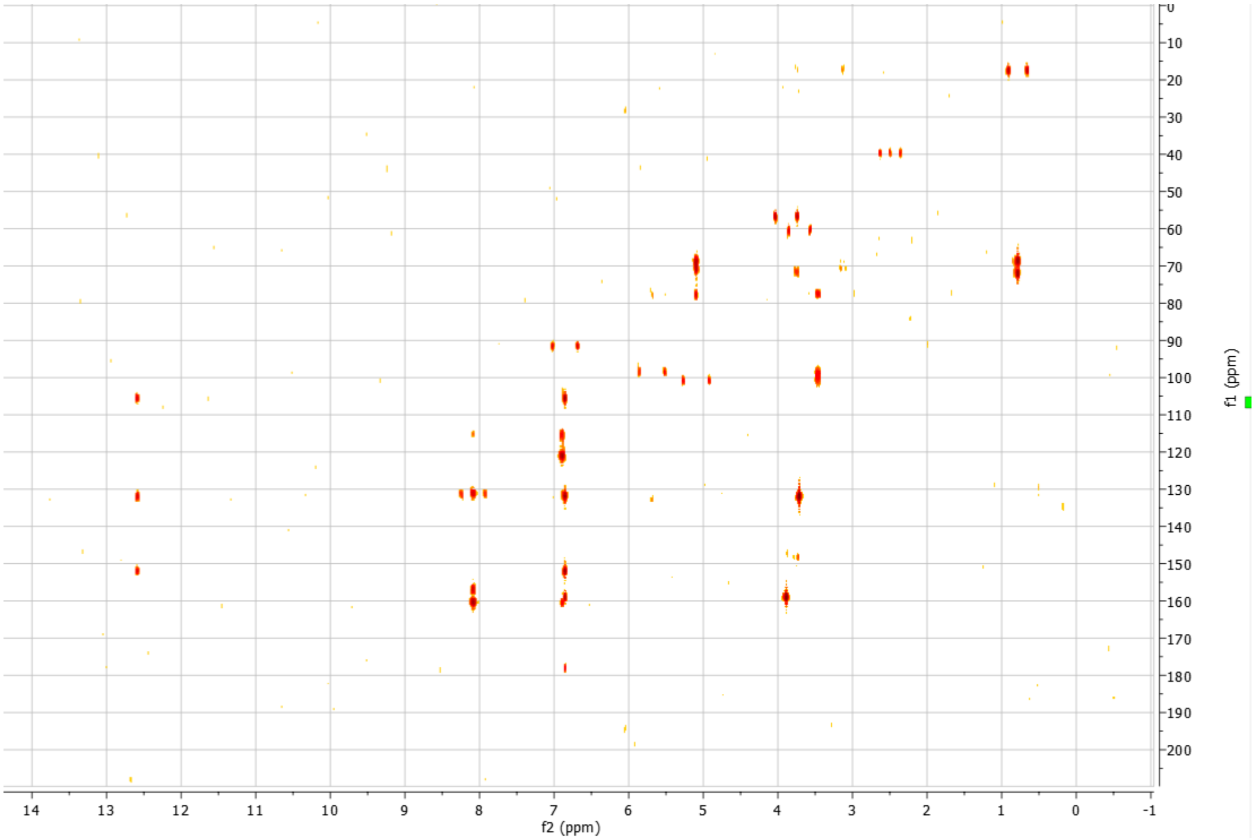

Figure S5. HMBC NMR spectrum compound **1.**

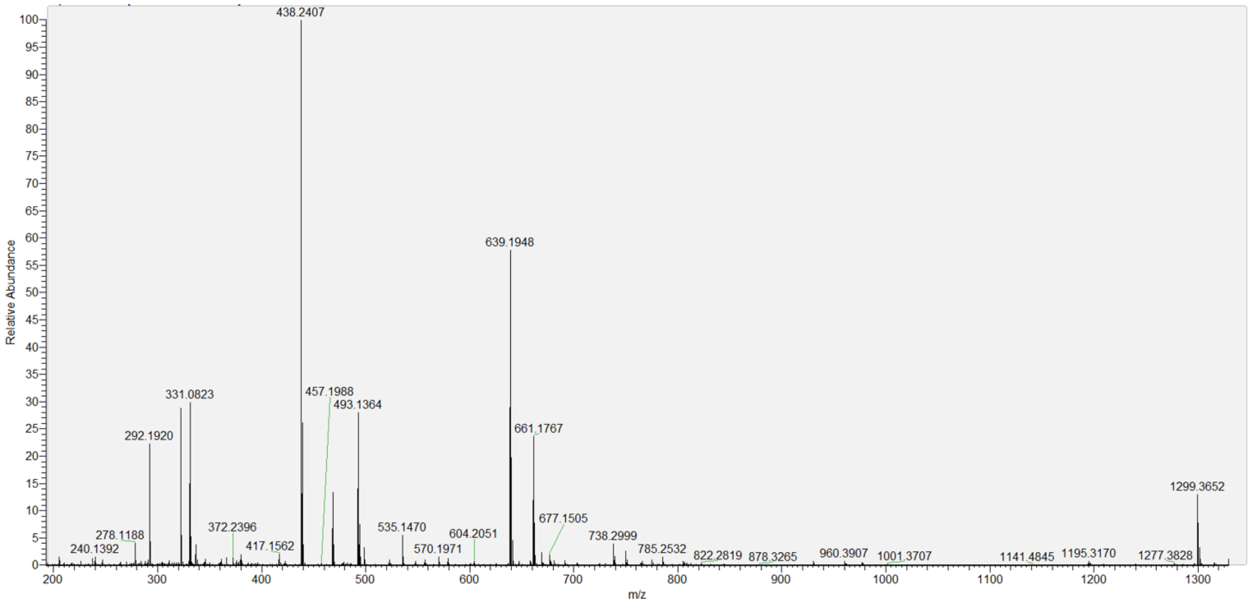

Figure S6. Positive HRESIMS of compound **1.**

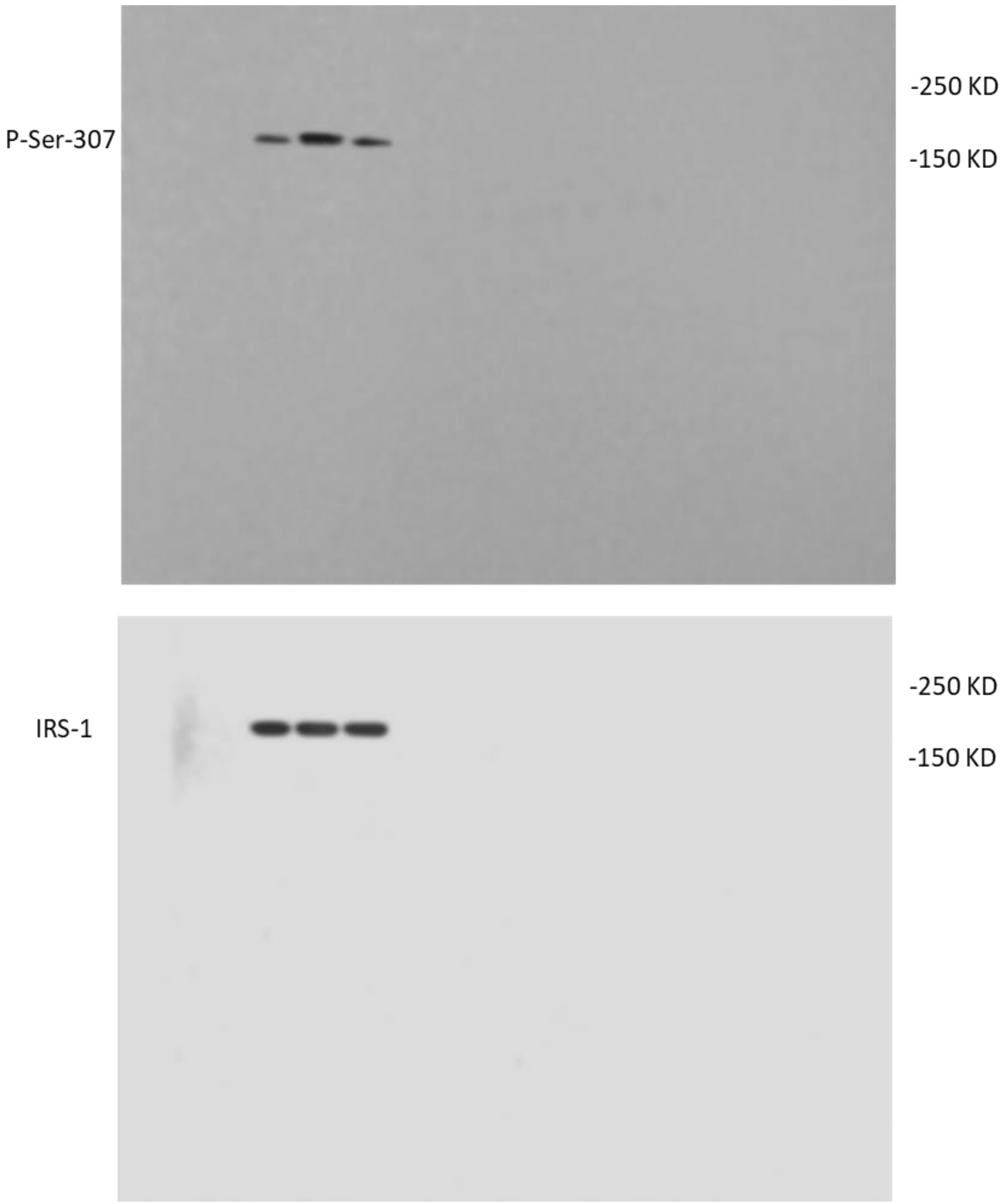
P-Ser-307
-250 KD
-150 KD
IRS-1
-250 KD
-150 KD

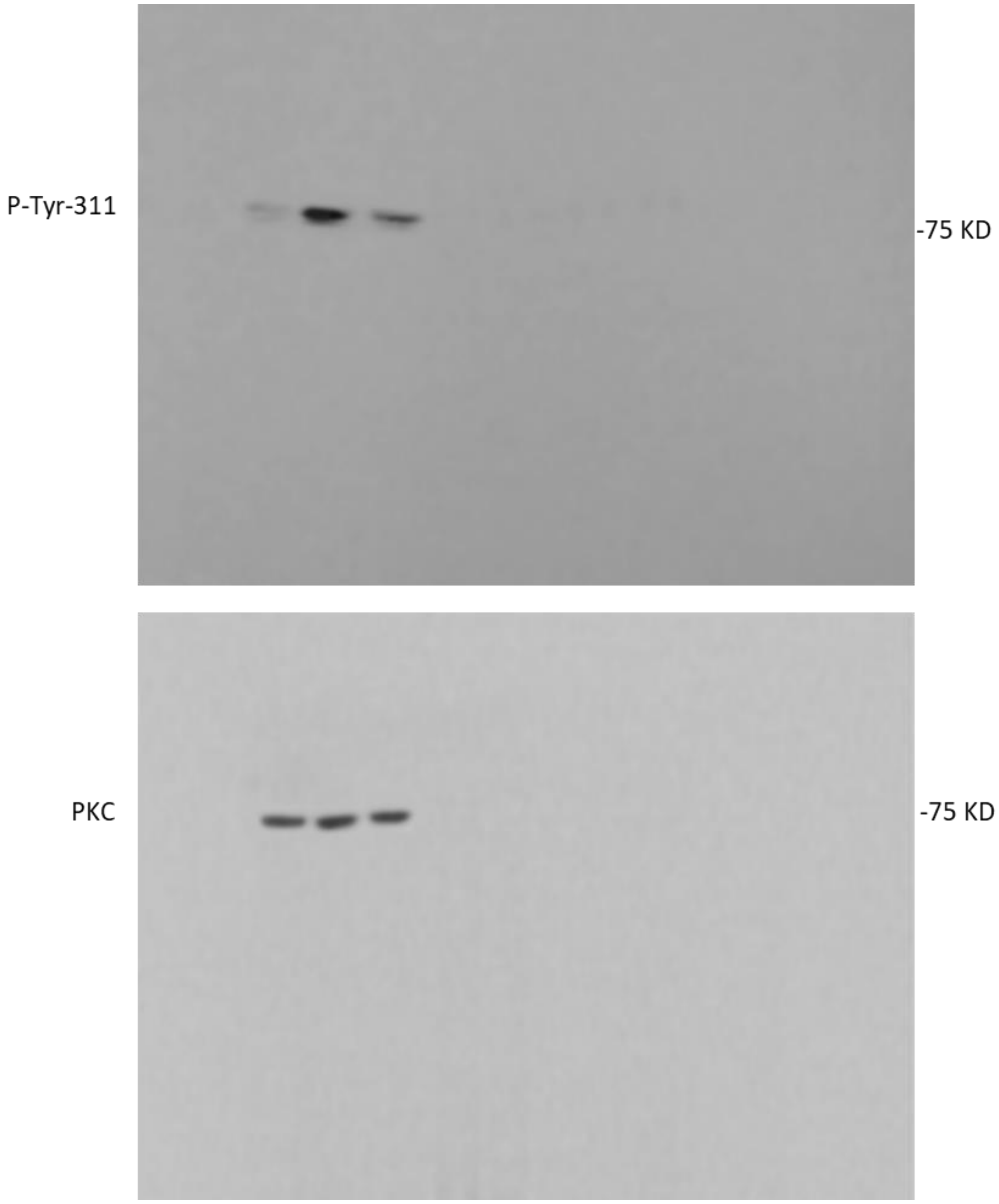
P-Tyr-311
-75 KD
PKC
-75 KD

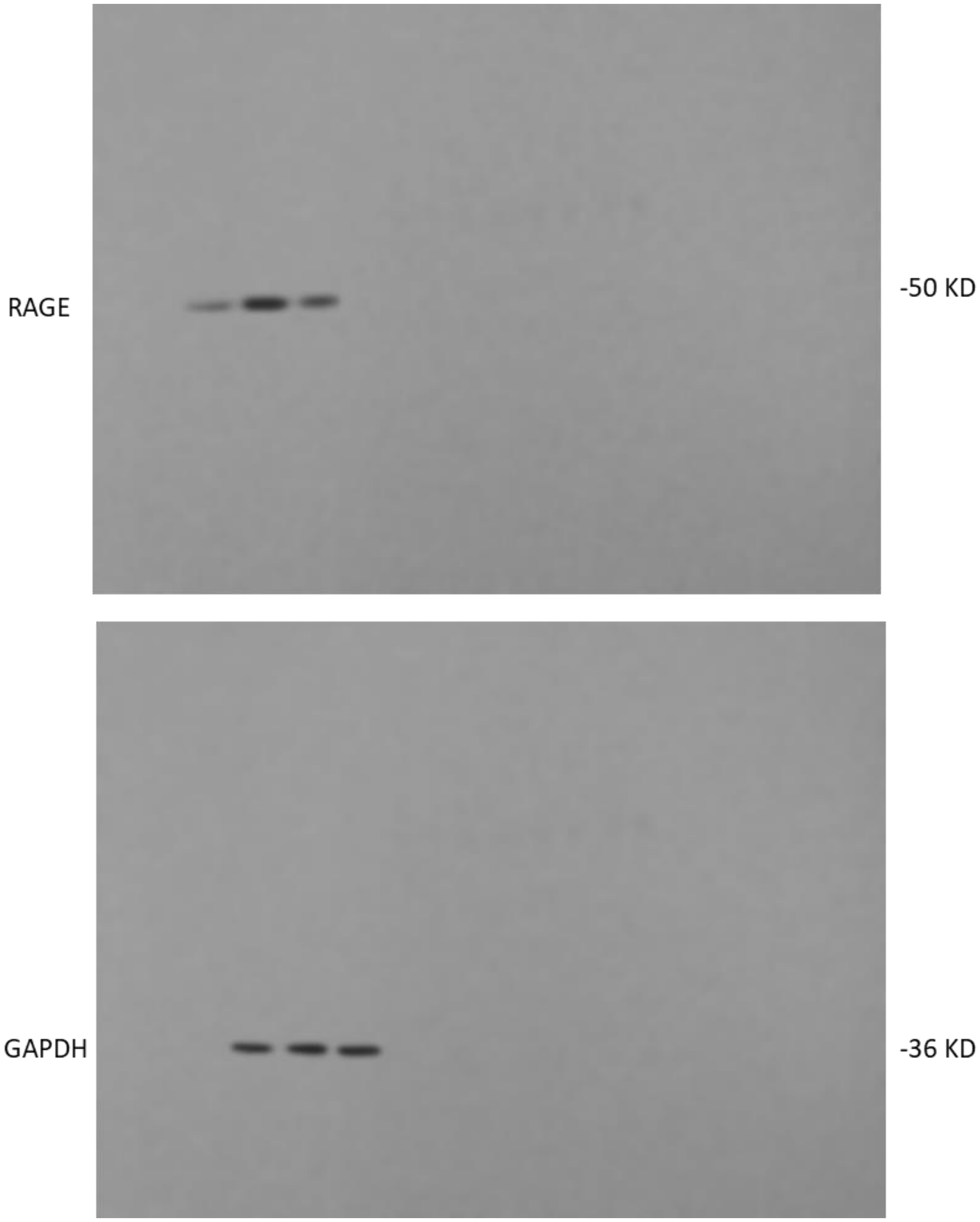

Figure S7. Western Blots images.

Table S1. Primers used for real time qPCR.

Gene	Protein	Forward primer	Reverse Primer
AGER	RAGE	5´-GGCAGACAGAGCCAGGAC-3’	5´-AGCACCCAGGCTCCAACT-3’
RELA	NF-kB (p65 subunit)	5’-ACTGTGTGACAAGGTGCAGAA-3’	5’-CACTTGTCGGTGCACATCA-3
